# Tocilizumab: Another medication related to osteonecrosis of the jaws? A case report and literature review

**DOI:** 10.3205/iprs000153

**Published:** 2021-04-14

**Authors:** Andreas Sakkas, Sebastian Heil, Steffen Kargus, Martin Rebel, Robert A. Mischkowski, Oliver C. Thiele

**Affiliations:** 1 Department of Oral, Maxillofacial and Facial Plastic Surgery, Ludwigshafen Hospital, Ludwigshafen, Germany; 2 Department of Pathology, Ludwigshafen Hospital, Ludwigshafen, Germany

**Keywords:** medication-related osteonecrosis of the jaw, tocilizumab, interleukin-6 receptor inhibitors, rheumatoid arthritis, osteomyelitis

## Abstract

**Introduction:** Medication-related osteonecrosis of the jaw (MRONJ) is a serious complication in patients receiving antiresorptive medication, such as bisphosphonates and denosumab, for different oncologic and non-oncologic diseases. Here, we report a case of MRONJ in a patient treated with tocilizumab, a humanized anti-interleukin-6 receptor antibody that effectively treats moderate to severe rheumatoid arthritis in adults.

**Case description:** A 45-year-old female patient diagnosed with severe rheumatoid arthritis, who had been undergoing intravenous tocilizumab therapy for three years without history of bisphosphonate use, was referred to our department. Four weeks previously, several teeth in the maxilla and mandible were removed under local anesthesia by her dentist. Two weeks after the extractions, she felt pain in both jaws. We diagnosed wound dehiscence and delayed healing of the alveolar bone after the tooth extractions. Digital volume tomography showed persistent dry alveolar sockets. The patient underwent surgical debridement of necrotic bone, and intravenous antibiotics were administered in hospital. Five months later, wound dehiscence reoccurred in the same regions. Histopathological analysis of bone biopsies revealed a diagnosis of MRONJ. Four months later, wound dehiscence occurred in the left maxillary alveolar ridge, and local bone resection was performed under antibiotic treatment. Twenty-four months after the last surgery, wound dehiscence had healed completely without signs of recurrence.

**Discussion:** Osteomyelitis of the jaw in patients treated with tocilizumab has not been reported often. This case confirms the potential role of this interleukin-6 receptor inhibitor in the pathogenesis of MRONJ and shows that patients who receive tocilizumab with MRONJ-like symptoms should be closely monitored. The pathomechanism of MRONJ under tocilizumab therapy remains unclear, so dental practitioners, maxillofacial surgeons, and rheumatologists should look for signs of MRONJ in patients receiving tocilizumab to prevent MRONJ onset.

## Zusammenfassung

**Einleitung:** Die Medikamenten-assoziierte Osteonekrose des Kiefers (MRONJ) ist eine schwerwiegende Komplikation bei Patienten unter antiresorptiver Therapie wie beispielsweise Bisphosphonaten oder Denosumab, die im Rahmen verschiedener onkologischer und auch nicht-onkologischer Therapien verabreicht werden.

Hier möchten wir von einer manifesten MRONJ bei einer Patientin berichten, die bei rheumatoider Arthritis mittels Tocilizumab, einem humanisierten, monoklonalen Antikörper gegen den Interleukin-6 (IL-6)-Rezeptor behandelt wurde.

**Fallbeschreibung:** Eine 45-jährige Patientin, die bei rheumatoider Arthritis seit drei Jahren intravenös Tocilizumab erhielt und keine Bisphosphonat-Anamnese aufwies, wurde zur Therapieübernahme in unserer Klinik vorgestellt. Vier Wochen zuvor waren durch den Hauszahnarzt in Lokalanästhesie multiple Zähne im Ober- und Unterkiefer entfernt worden. Zwei Wochen nach dem Eingriff beklagte die Patientin Schmerzen in beiden Kiefern. Wir sahen dehiszente Wundverhältnisse und eine deutlich verzögerte Heilungstendenz der Extraktionsalveolen. In der Digitalen Volumentomographie (DVT) zeigten sich persistierende Alveolen. Unter stationären Bedingungen führten wir nach intravenöser antibiotischer Aufsättigung die Dekortikation mit Entfernung der Knochennekrosen durch. Histopathologisch bestätigte sich der Verdacht der MRONJ.

Nach fünf ereignisfreien Monaten stellte sich die Patientin mit erneuter Wunddehiszenz in beiden Kiefern vor. Nach weiteren vier Monaten konservativer Therapie verblieb eine Wunddehiszenz im linken Oberkiefer. Hier erfolgte erneut unter antibiotischer Abschirmung die lokale Dekortikation mit plastischer Deckung. Insgesamt 24 Monate nach diesem letzten chirurgischen Eingriff war die Patientin beschwerdefrei und es lagen reizfreie Wundverhältnisse vor. Ein Anhalt für ein erneutes Rezidiv bestand nicht.

**Diskussion:** Eine Osteomyelitis des Kiefers bei Patienten unter Tocilizumab ist bisher äußerst selten berichtet.

Dieser Fallbericht bestätigt die möglicherweise wichtige Rolle von Interleukin-6 (IL-6)-Rezeptor-Inhibitoren in der Pathogenese der MRONJ und zeigt, dass diese Patienten engmaschig kontrolliert werden und insbesondere bei Symptomen einer MRONJ einer entsprechend spezialisierten Klinik zugewiesen werden sollten.

Der Pathomechanismus einer MRONJ unter Tocilizumab-Therapie bleibt unklar, so dass wir empfehlen, dass Zahnärzte, MKG-Chirurgen und Rheumatologen für das Auftreten einer MRONJ in diesem Patientengut sensibilisiert sind.

## Background

In 2014, the American Association of Oral and Maxillofacial Surgeons (AAOMS) recommended a nomenclature change from bisphosphonate-related osteonecrosis of the jaw (BRONJ) to medication-related osteonecrosis of the jaw (MRONJ) because a growing number of osteonecrosis cases were associated with other antiresorptive and antiangiogenic drugs [1], [2]. In 2014, the AAOMS defined MRONJ as “the presence of exposed necrotic bone or bone that can be probed through an intraoral or extraoral fistula in the maxillofacial region that has persisted for longer than eight weeks, occurring in patients undergoing treatment with antiresorptive or antiangiogenic agents with no history of radiation therapy or obvious metastatic disease to the jaws” [1]. The clinical manifestation of MRONJ is multifactorial and mainly depends on the duration of antiresorptive therapy, oral or intravenous application, combined treatment with corticosteroids or diverse chemotherapies, and local risk factors such as surgery-related tissue trauma and oral health of the patient [3], [4].

Except classic bisphoshonates, the role of humanized monoclonal antibodies such as denosumab, bevacizumab, and adalimumab, as well as chimeric monoclonal antibodies such as rituximab in MRONJ pathogenesis has been described in several publications [2], [5], [6], [7], [8], [9], [10], [11], [12], [13], [14], [15], [16], [17], [18], [19], [20]. Targeted therapies have been developed to control the growth and survival of malignant cells by interfering with specific molecular routes involved in carcinogenesis. However, recent publications investigating the safety of cancer target therapies have indicated that oral complications are still important in contemporary cancer treatment [2]. Since the receptor activator of nuclear factor kappa-B ligand (RANKL) and TNF-α have similar biological features and RANKL inhibition by denosumab is associated with MRONJ, interleukin-6 receptor inhibition may also be associated with MRONJ [2].

Tocilizumab is a humanized monoclonal antibody against the interleukin-6 receptor. Interleukin-6 plays a critical role in naïve T cell differentiation into Th17 cells in cooperation with transforming growth factor-β. Interleukin 6 is involved in the development of immunological and inflammatory reactions. Autoimmune diseases like rheumatoid arthritis are associated with abnormally high interleukin-6 levels. Tocilizumab binds soluble and membrane-bound interleukin-6 receptors, preventing interleukin-6 from exerting its pro-inflammatory effects. Neutralizing interleukin-6 with inhibitors such as tocilizumab reduces the progression of autoimmune diseases [11], [12]. Tocilizumab can be used to treat moderate to severe rheumatoid arthritis and systemic juvenile idiopathic arthritis, mostly combined with methotrexate. Encouraging results of tocilizumab use in the treatment of COVID-19 affected patients mainly in China due to the recent coronavirus pandemic have also been reported [21], [22]. 

As well as its positive anti-inflammatory activity, a series of negative side effects have been associated with tocilizumab therapy, including upper respiratory tract infections, nasopharyngitis, headache, hypertension, and elevated total cholesterol. The risk of medication-related osteonecrosis of the jaw in patients taking tocilizumab have been well investigated to date. The lack of sufficient clinical reports means the frequency and severity of MRONJ caused by interleukin-6 receptor inhibitor cannot be reliably assessed [13], [14], [15].

Three case reports have described the clinical occurrence of MRONJ in patients treated with tocilizumab [13], [14], [15]. Here, we present a new case of osteomyelitis in the maxilla and mandible of a patient affected by severe rheumatoid arthritis that was aggravated by tocilizumab and developed into osteonecrosis. MRONJ has not been assessed in patients treated with interleukin-6 receptor inhibitors so far. The additional case of MRONJ related to tocilizumab use presented here suggests that immunosuppressive medication may cause MRONJ in patients who have not previously been exposed to bisphosphonates.

## Case description

A 45-year-old female patient presented to our craniomaxillofacial surgery department (Ludwigshafen hospital in Germany) in March 2017 with persistent pain in the maxilla and mandible after multiple tooth extractions.

The patient’s general medical history revealed that she had been diagnosed with severe rheumatoid arthritis, which had been treated with monthly intravenous infusions of 162 mg tocilizumab and weekly subcutaneous infusions of 15 mg methotrexate for the last three years. Comorbidities included latent hypothyreosis, insulin-dependent diabetes mellitus type II, chronic renal insufficiency II, and arterial hypertension. No history of smoking or alcohol abuse was reported. The patient did not have history of bisphosphonate or corticosteroid therapy.

**Figure 1:**
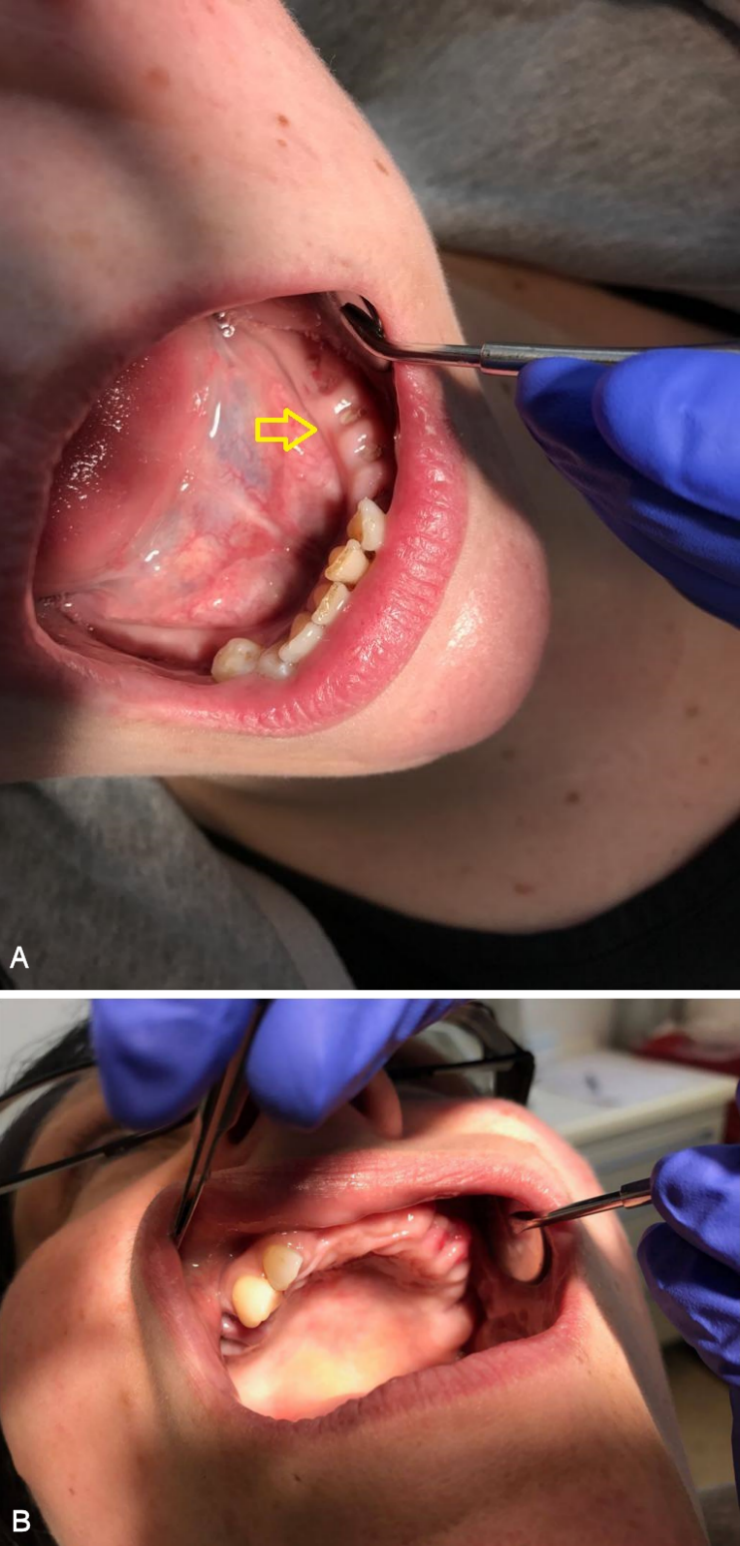
Patient’s clinical features 4 weeks after tooth extractions. Intraoral examination revealed persistent alveolar sockets and local nfection on the left side of the mandible (A). Necrotic bone exposure and persistent alveolar sockets on the right and left side of the maxilla (B). The lesion was classified as stage 2 medication-related osteonecrosis of the jaw according to the American Association of Oral and Maxillofacial Surgeons staging system [1].

Four weeks before being referred to our department, several of the patient’s teeth were extracted from the maxilla and mandible under local anesthesia by her dentist. No intraoperative complications were reported during the extractions. Two weeks after surgery, the patient felt severe pain in both jaws. The pain persisted despite appropriate analgesic therapy, and the patient came to our department four weeks after surgery. An intraoral clinical examination revealed infected, dry alveolar sockets in the maxilla and mandible (regions #15-14, #11-27, #33-36 and #45-46) without signs of mucosal abscess or purulent discharge (Figure 1 (Fig. 1)). Moderate marginal periodontitis of the remaining teeth was also diagnosed, with 2 mm attachment loss, 2–3 mm probing depth, and a horizontal bony defect. The patient was able to swallow and open her mouth normally without pain. Extraorally, no lymphadenopathy or other alterations were evident. 

**Figure 2:**
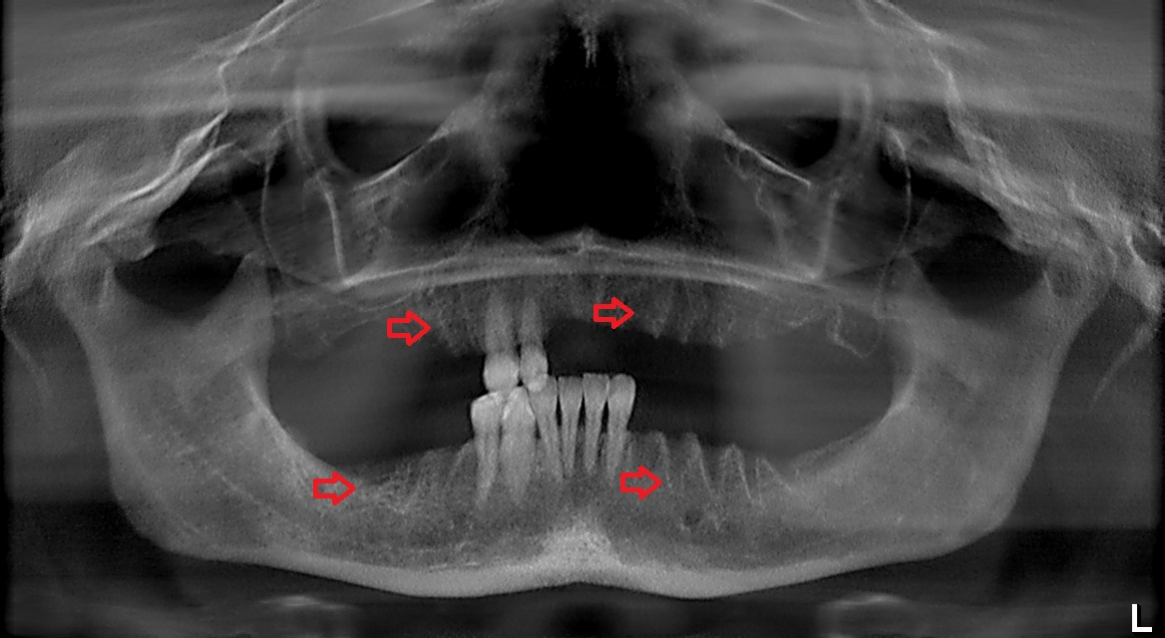
Panoramic radiograph examination (performed in March 2017) revealed osteonecrosis, osteolysis, and subperiosteal bone deposition. Arrows show persistent unremodeled extraction sockets at surgical sites in the mandible and maxilla.

**Figure 3:**
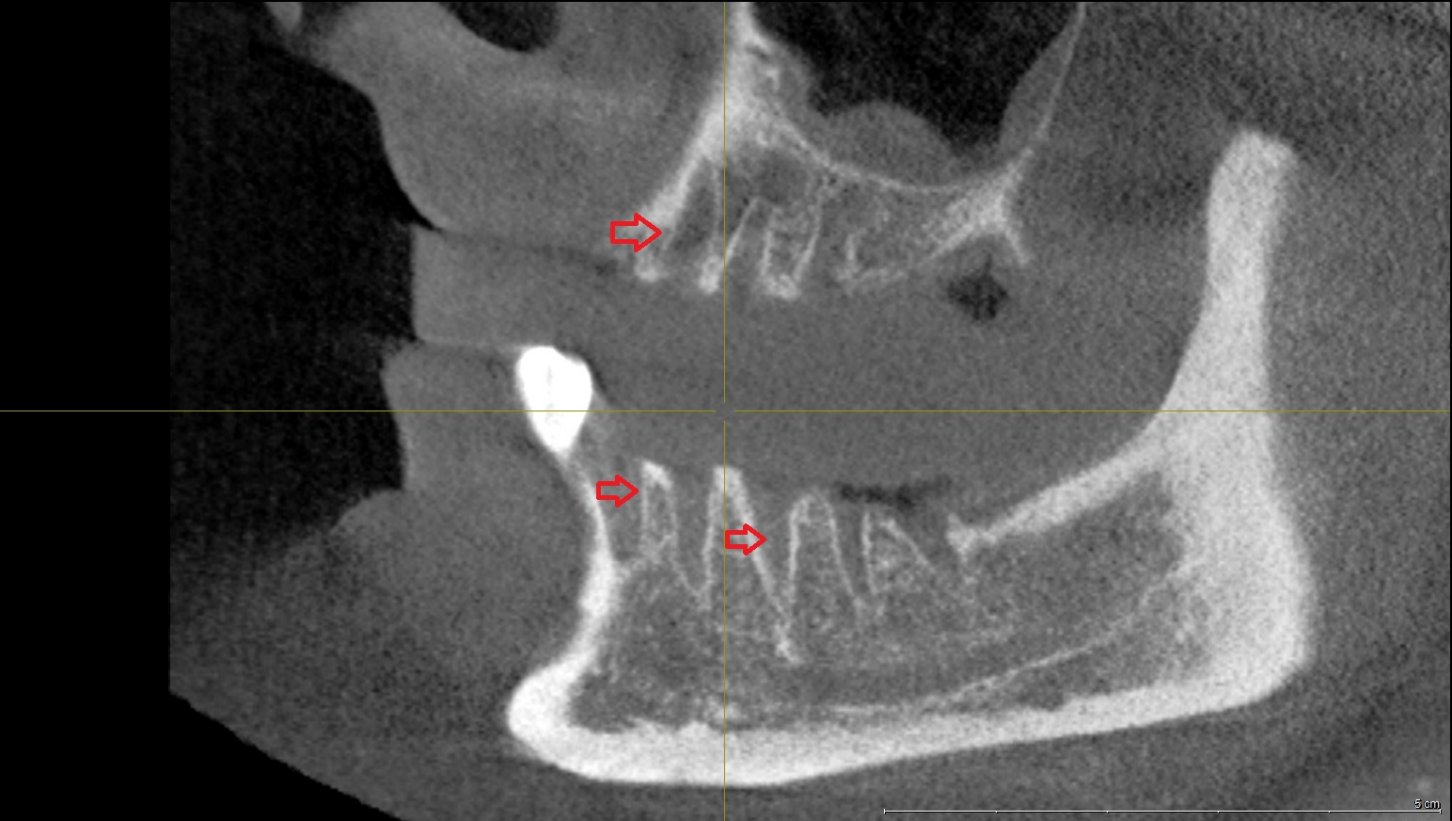
Digital volume tomography (performed in March 2017). Sagittal view shows persistent bony trabecular architecture without bony lysis, indicating classical radiologic signs of persistent dry alveolar sockets. Arrows indicate the unremodeled alveolar sockets.

A panoramic radiograph obtained on the day of her visit on March 2017 revealed regions of osteosclerosis, subperiosteal bone deposition at the extraction sites, and persistent unremodeled bone in the alveolar sockets surrounded by periapical hypermineralized lines (Figure 2 (Fig. 2)). A generalized periodontitis combined with tooth loss and horizontal bone loss was also diagnosed radiologically. A digital volume tomography in sagittal view showed persistent bony trabecular architecture and absence of bony lysis – the classical radiologic signs of “persistent dry alveolar sockets” (Figure 3 (Fig. 3)).

Based on these findings, a diagnosis of MRONJ with exposed necrotic bone was suspected. We considered the bony lesion to be stage 2 MRONJ based on the AAOMS severity staging system; necrotic bone was exposed and infected (as evidenced by pain and erythema at the sites of extraction) without purulent drainage [1].

**Figure 4:**
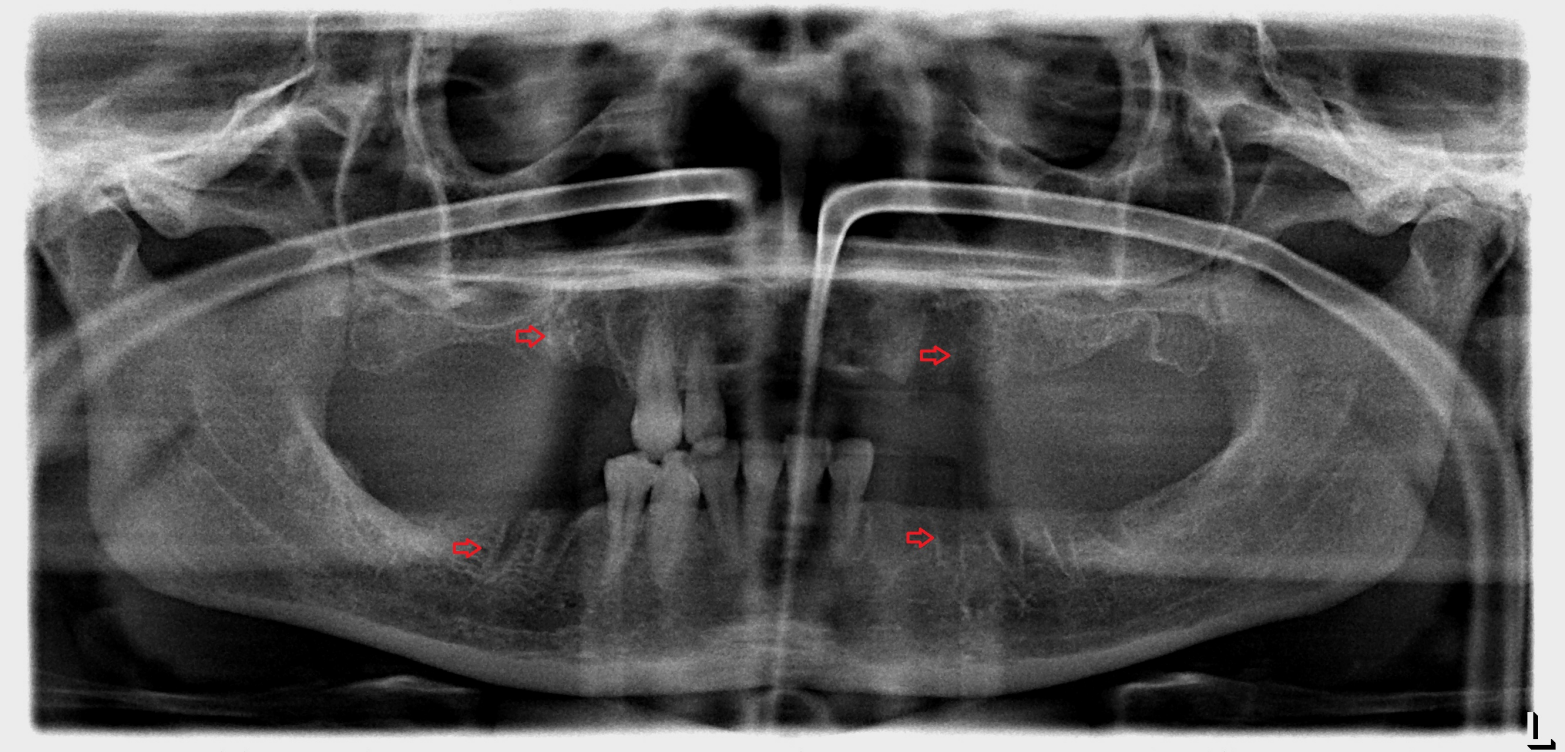
Postoperative panoramic radiograph (performed in March 2017) after surgical decortication, showing diffuse bone resorption of the mandible in the region of alveolar exposure, and the remaining vital alveolar bone.

The patient underwent surgical debridement of the necrotic alveolar bone and local mucosal closure under general anesthesia. The postoperative orthopantomograph showed reduced alveolar bone height without sequesters after surgical revision (Figure 4 (Fig. 4)). Ampicillin/sulbactam 3 g (Unacid^®^, Pfizer Pharma GmbH, Germany) were administered intravenously three times a day, and appropriate analgesia was administered for 7 days under hospitalization. A nasogastric tube was used for continuous feeding during this period to avoid soft tissue manipulation at the surgical sites. During hospitalization, the surgical sites were treated with chlorhexidine mouth rinses (0.12%) under continuous antibiotic therapy. After 7 days, the patient was discharged home and followed-up daily in our outpatient clinic. The local treatment consisted of irrigating the mouth with chlorhexidine and appropriate mouth hygiene. After hospitalization, no more antibiotics were administered. On review, there was a considerable improvement in her physical condition; her systemic symptoms had objectively subsided and the pain reduced, decreasing the analgesic intake. Two weeks post-operation, we noticed a complete remission of the clinical symptoms, without further dehiscence of the alveolar bone.

In August 2017, following an uneventful 5-month period, the patient presented in our department with recurring pain. Intraoral dehiscence of the alveolar bone and accumulated food debris in the left side of the maxilla and mandibular region were detected. An initial conservative therapeutic approach of oral chlorhexidine rinses (0.12%) and oral antibiotics was carried out for 5 days to try and achieve secondary wound closure without bone debridement. The patient was monitored closely. After this conservative treatment, avital alveolar bone exposure was still present without pus secretion and intraoral wound healing was uneventful. The treatment with tocilizumab was not interrupted during treatment in our clinic.

**Figure 5:**
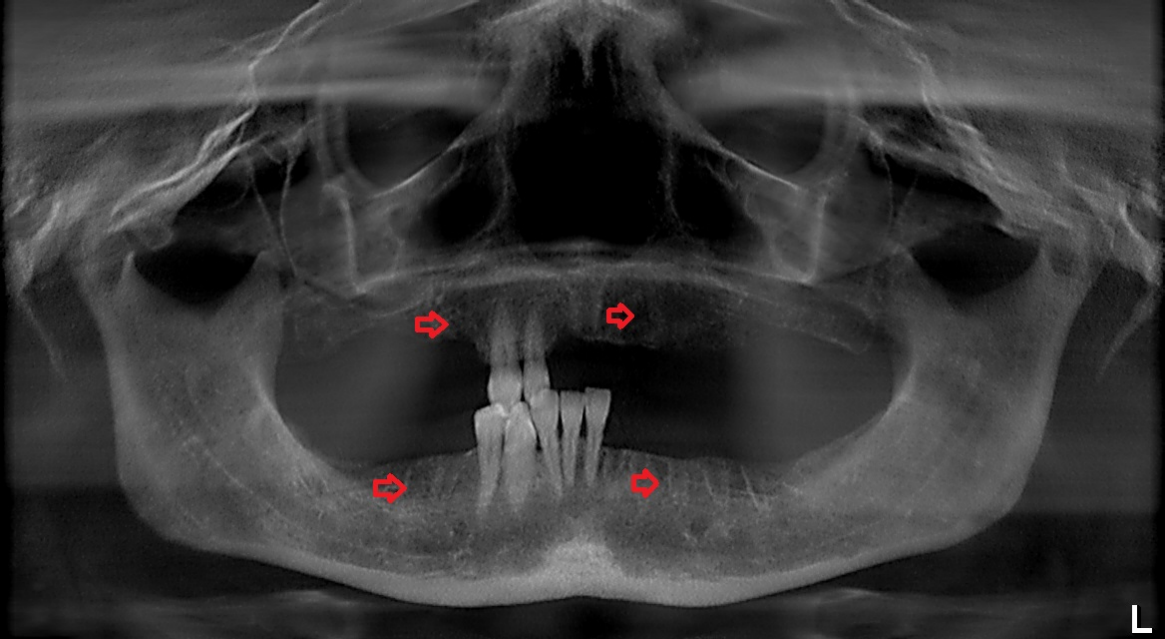
Panoramic radiograph (performed in August 2017) after the second alveolar decortication, 5 months after first surgical approach, presenting the remaining vital alveolar bone of the mandible and maxilla.

**Figure 6:**
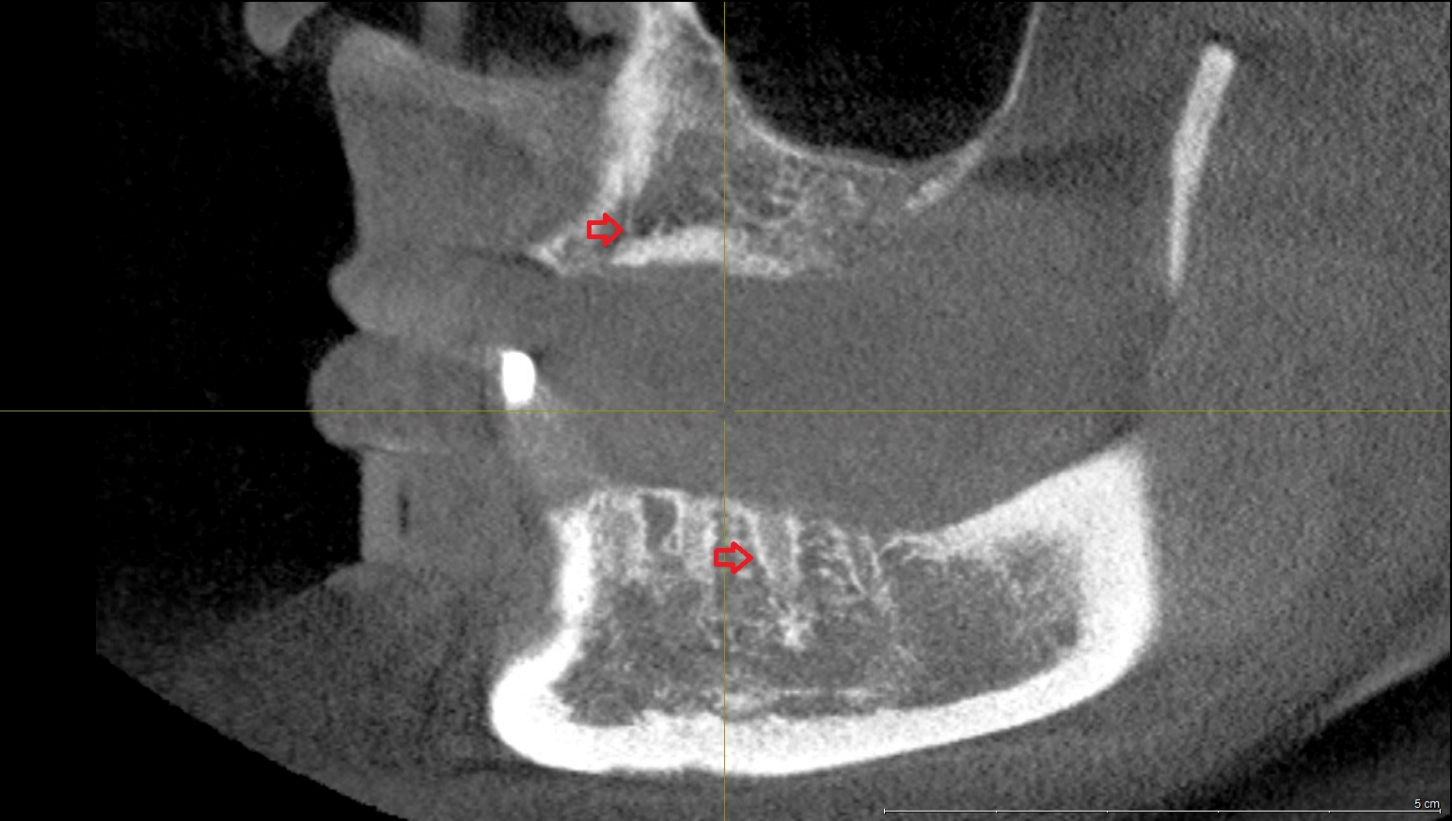
Sagittal view of the digital volume tomography (performed in August 2017) after surgical decortication. Arrows show persistent bony trabecular architecture without signs of osteonecrosis. Clinically, no signs of wound dehiscence or bone exposure were detected.

The clinical and radiographic examination revealed mandibular osteonecrosis and the patient underwent surgery under general anesthesia. This involved partial decortication of the infected maxilla and mandible (the residual anterior-posterior alveolar process and the bicortical plate exposed to vital bone) with preservation of the alveolar inferior nerve and the basal cortical margin. After resection, residual infected and necrotic tissues were removed from the remaining avital bone surface by a rotating burr and diamond burr in an attempt to prevent MRONJ recurrence. The exposed alveolar inferior nerve was protected from all cutting instruments during surgery. The wound was closed after periosteal releasing incisions, to achieve a tension-free adaption of the soft tissues. As described earlier, an adjuvant cycle of antibiotics was administered and a nasogastric tube was fitted under hospitalization for 5 days. A postoperative orthopantomograph was performed in August 2017 after surgical decortication showed vital alveolar bone (Figure 5 (Fig. 5)). The sagittal view of the digital volume tomography showed persistent bony trabecular architecture without signs of osteonecrosis (Figure 6 (Fig. 6)). No neural alterations were detected on the day after surgery. Sutures were removed 2 weeks after surgery and no intraoral wound dehiscence or new bone exposure was detected.

**Figure 7:**
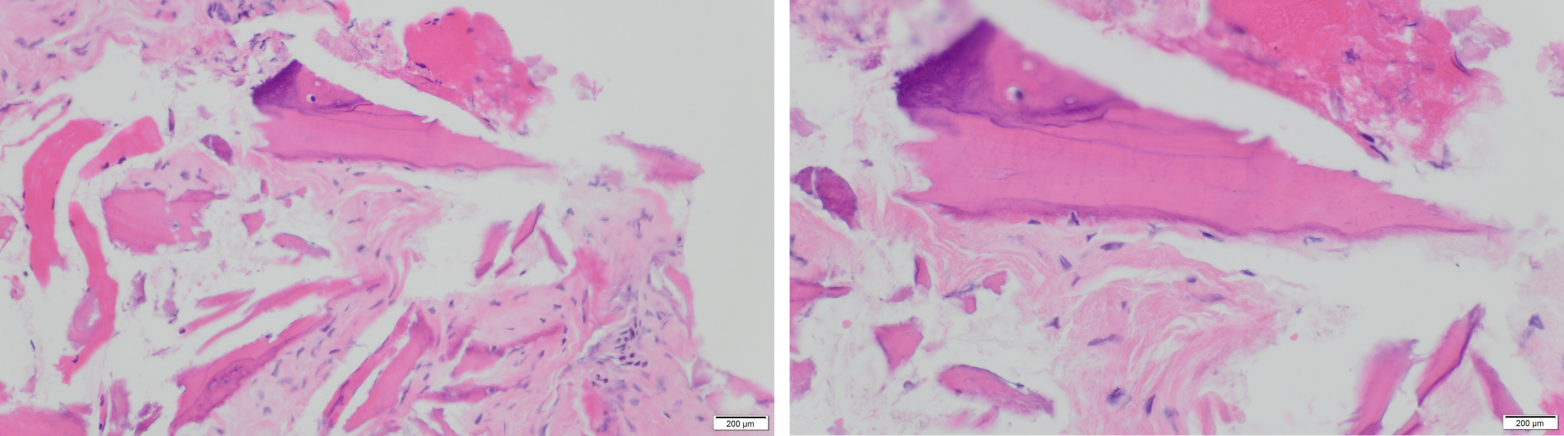
Histopathological analysis of the decalcified samples stained with hematoxylin-eosin shows areas of bone necrosis with inflammatory cell infiltration and several basophilic bacterial colonies, empty Haversian canals without residual osteocytes/osteoblasts, and reduction of Haversian blood vessels, thus confirming the clinical diagnosis of MRONJ (left: 200× magnification, right: 400× magnification). Areas of remaining vital alveolar bone are also shown.

The surgical specimen was fixed in neutral-buffered formalin and sent to the pathological anatomy department of our hospital, where it was decalcified in formic acid, embedded in paraffin, sectioned at 4-µm thickness, and stained with hematoxylin-eosin. The histopathological analysis of the decalcified samples showed areas of bone necrosis with inflammatory cell infiltration, several basophilic bacterial colonies, empty Haversian canals without residual osteocytes/osteoblasts, and fewer Haversian blood vessels, thus confirming the clinical diagnosis of MRONJ (Figure 7 (Fig. 7)).

On December 2017, 4 months after clinical recurrence and surgical treatment, the patient returned to our department with recurring pain in the posterior maxilla. Bone exposure and 2× 3 mm wound dehiscence in the left maxillary alveolar ridge (region #26-27) were observed, without signs of abscess. We performed a surgical revision with rotating and diamond burrs to remove necrotic bone tissues and tension-free wound closure under local anesthesia. Oral antibiotics were again administered for 5 days.

Twenty-four months after the last surgical treatment, no further complications or symptoms of recurrence were detected.

## Discussion

The 2014 AAOMS Position Paper outlined that MRONJ can be diagnosed if the following are present [1]:

1. Current or previous treatment with antiresorptive or antiangiogenic agents
2. Exposed bone or bone that can be probed through an intraoral or extraoral fistula in the maxillofacial region that has persisted for longer than 8 weeks
3. No history of radiation therapy to the jaws or obvious metastatic disease of the jaws

MRONJ caused by intravenous and oral bisphosphonates has been extensively researched over the last years. Inhibitors of RANKL (denosumab), angiogenesis (bevacizumab and rituximab), tyrosine kinase receptors (sunitinib), and TNF (adalimumab) have been related to MRONJ in published case reports. This prompted an international nomenclature change from BRONJ to MRONJ [1], [19], [20], [23], [24], [25], [26]. Histologically, MRONJ appears similar to osteomyelitis, and it is not clear whether infection is the cause or consequence of bone exposure.

Interleukin-6 is a cytokine involved in immunological and inflammatory reactions [12], [27], [28]. Interleukin-6 mediates a wide spectrum of biological activities including activation of T cells, differentiation of B cells, induction of acute phase reactants, proliferation of fibroblasts, and damage to cartilage and joints. The role of interleukin-6 as an anti-inflammatory cytokine is mediated through its inhibitory effects on TNF-α and interleukin-1, and activation of interleukin-1 receptor antagonist and interleukin-10 [27], [28]. Autoimmune diseases like rheumatoid arthritis are associated with abnormally high interleukin-6 levels. Tocilizumab, a humanized monoclonal antibody specific for the interleukin-6 receptor, binds soluble and membrane-bound interleukin-6 receptors, thereby preventing interleukin-6 from exerting its pro-inflammatory effects [27], [28]. Tocilizumab was approved for use in the United States in 2010 and current indications include refractory rheumatoid arthritis and polyarticular and systemic forms of juvenile idiopathic arthritis. Tocilizumab is considered a disease-modifying antirheumatic drug that improves signs and symptoms of the disease and reduces destruction of cartilage and tissue. Tocilizumab is given by intravenous infusion every 4 weeks in doses of 4–12 mg/kg depending on the indication and body weight. It is applied mostly in combination with methotrexate, if other antirheumatic drugs and TNF-α blockers have proven to be ineffective or were not tolerated [11], [12]. It can also be used as a monotherapy by patients who do not tolerate methotrexate. Tocilizumab slows down the progression of arthritis and can improve physical function of patients. The treatment of systemic juvenile idiopathic arthritis with tocilizumab has also been established [29]. Tocilizumab is currently available in vials of 20 mg/mL under the brand name RoActemra^®^ (Hoffmann-La Roche AG, Switzerland).

Recent scientific reports declare the intravenous administration of tocilizumab in the treatment of the COVID-19 related acute respiratory insufficiency with encouraging results [30], [31], [21], [22]. Due to an overproduction of proinflammatory cytokines and especially IL-6, a large number of COVID-19 affected patients in critical situation experience a “cytokine storm” [31]. This storm contributes to increased disease severity as well as aggravation of prognosis, and tocilizumab contributes to symptom improvement by reducing the IL-6 expression associated inflammation [31]. However, further studies are needed to validate the ability of tocilizumab to restore T cell counts in COVID-19 patients. 

The aim of this article was to describe an original case of tocilizumab-related MRONJ in a patient affected by severe rheumatoid arthritis. This patient was receiving an intravenous infusion of 162 mg tocilizumab every 4 weeks, combined with 15 mg methotrexate subcutaneously every week.

Two cases of MRONJ following the administration of tocilizumab have been reported since 2012. However, in these cases, bisphosphonates or other antiresorptive drugs were administered concurrently. The first case, reported by Ebker et al. in 2013, was a 74-year-old female patient with rheumatoid arthritis treated with infliximab and tocilizumab [13]. The patient presented with a left-sided perimandibular abscess after extraction of the left second lower premolar. There were signs of advanced BRONJ with exposed bone and extraoral fistula formation in the left submandibular region three months after extraction. The panoramic radiograph showed a persistent extraction socket surrounded by periapical hypermineralized lines in the left mandible. The patient was receiving bisphosphonates for osteoporosis as well as methotrexate and prednisolon for rheumatoid arthritis, therefore the role of tocilizumab in the onset of MRONJ could not be confirmed. However, Ebker et al. did point out that tocilizumab may be (at least partly) responsible for the pathomechanism of MRONJ. Another report in 2013 described mandibular osteomyelitis in a 60-year-old Japanese woman diagnosed with rheumatoid arthritis following tocilizumab therapy [14]. The authors described mandibular osteomyelitis as a potential complication of tocilizumab treatment, highlighting its use as a risk factor in oral surgery [14]. The clinicians diagnosed a swelling of the right submandibular region extended to the cervical and axillary lymph nodes as well as a right lower gingival ulcer with necrotic bone and intraoral pus discharge. They proceeded with wide bone debridement and resection of the necrotic tissues under antibiotics. However, methotrexate and sodium risedronate hydrate were also administered, so the role of tocilizumab in the pathogenesis of MRONJ could not be confirmed (Table 1 (Tab. 1)).

The first case of MRONJ in a patient treated with tocilizumab for rheumatoid arthritis without treatment with bisphosphonate or other MRONJ-related drugs was reported by Bindajhil et al. in 2018 [15]. They presented the case of a 71-year-old female with intermittent spontaneous bleeding from mandibular left gingiva for 6 months after multiple extractions (Table 1 (Tab. 1)). The panoramic radiograph revealed massive bone destruction and large radiolucency at the extraction sites, extending inferiorly up to the superior cortex of the mandibular canal. Cone beam tomography showed expanded lucency in the body of the left mandible, with erosion of the buccal cortical bone, significant loss of bone structure, and reduced height of the alveolus. The radiographic and histological features after surgical treatment led to the diagnosis of MRONJ [15].

Based on the expanded therapeutic potential of tocilizumab due to the current COVID-19 pandemic and regarding its clinical relevance in oral surgery procedures according to the above mentioned clinical reports, Imaculada de Queiroz Rodrigues et al. evaluated the effect of pretreatment with tocilizumab on bone remodeling in rats after tooth extraction [31]. In this study, the mandible of 48 rats was clinically and histomorphologically evaluated after intravenous administration of tocilizumab and extraction of the first lower left molar. According to its results, reduced osteoclast count, increased histological signs of infection, myeloid suppression and induction of response in neutrophil infiltration of the alveolar mucosa were demonstrated. The suppression of IL-6 in rats is likely to contribute significantly to inflammatory alveolar bone destruction [31]. 

In the present case, maxilla and mandibular osteonecrosis developed after tooth extractions under tocilizumab treatment, without a history of bisphosphonate therapy but with concomitant methotrexate treatment. The persistent uneventful bone and mucosal healing after the extractions led us to diagnose MRONJ. The bony lesion was defined as stage 2 MRONJ according to the AAOMS staging system because the exposed necrotic bone was infected (as evidenced by pain and erythema at extraction sockets) without purulent drainage [1]. Radiological signs included osteosclerosis, cortical disruption, osteolysis, subperiosteal bone deposition, and thickening of lamina dura. Osteosclerosis is due to mineralization without balanced bone resorption; extraction sockets persist because bone remodeling is inhibited after tooth removal [17]. Delayed healing after tooth extraction and persistence of pain in combination with clinical surface irregularity and persistent sockets pointed to a suspected diagnosis of tocilizumab-related MRONJ.

The patient received intravenous antibiotic cycles during hospitalization, after which she underwent surgery twice, involving debridement of the lysed alveolar bone after her clinical symptoms recurred. Histopathological analysis of the surgical specimen confirmed the diagnosis of MRONJ. To date, the potential role of tocilizumab in the pathomechanism of MRONJ has not been well investigated [13], [14], [15]. However, we have noticed that tocilizumab may facilitate infectious healing complications in patients undergoing oral surgery because of its immunosuppressive effects. This case report together with other published cases of tocilizumab-related osteonecrosis of the jaw reinforces the view that tocilizumab contributes to the onset of jaw osteonecrosis. Although bisphosphonates and bevacizumab may also contribute to jaw osteonecrosis, it is too early to compare the time of onset, clinical progress, and outcomes of bisphosphonate- and bevacizumab-related jaw osteonecrosis.

We recognize several limitations to this case report. Tocilizumab was not the only treatment administered to our patient (the chemotherapy drug and immune suppressant methotrexate was also given), so we cannot draw valid conclusions regarding the role of tocilizumab in MRONJ pathogenesis. Further prospective studies with sufficient patient numbers and a standardized evaluation protocol are needed to confirm whether tocilizumab treatment is related to MRONJ. Patient and medication factors, such as treatment duration, concomitant therapies, diabetes mellitus or other autoimmune diseases, presence of marginal or apical periodontitis before treatment, and patient mouth hygiene should also be investigated to fully define the role of tocilizumab in MRONJ pathogenesis. Furthermore, it remains unknown to what extent tocilizumab contributes to MRONJ after treatment is discontinued. For example, the effect of denosumab on bone metabolism dissipates after 6 months because it is not retained in the bone, unlike bisphosphonates [18], [19]. The effect of interleukin-6 inhibitors such as tocilizumab on bone metabolism is probably explained by temporal interactions with their receptor. This suggests that tocilizumab only causes MRONJ when the patient is either taking the drug or has received the drug in the past few months. 

In summary, considering the nature of case reports like ours and the current lack of prospective studies, we can only assume causality between interleukin-6 inhibitors such as tocilizumab and MRONJ. With the promising future perspectives of tocilizumab use in immunoinflammatory diseases and in COVID-19 treatment, our clinical report as well as the research of Imaculada de Queiroz Rodrigues et al. [31] and previous case reports showed that in the clinical setting, surgeons need to pay attention to side effects of tocilizumab, especially due to increased risk of postsurgical infection. This clinical case recommends a dental examination before prescribing tocilizumab as well as periodic dental checkups during treatment. Although imaging is essential to evaluate the extent of the disease and to assess the treatment response, obtaining accurate and detailed medical history is also critical for MRONJ diagnosis.

## Conclusion

We reported a case of jaw osteonecrosis in a patient following tocilizumab therapy. Future prospective studies that consider other confounding factors, medical comorbidities, and concomitant medication are needed to determine the pathophysiology and occurrence of MRONJ in patients treated with tocilizumab. A regular dental checkup before and during tocilizumab administration is strongly recommended to prevent MRONJ recurring or to detect lesions at earlier stages.

Furthermore, the number of medications related to MRONJ onset is increasing; therefore, when prescribing drugs such as tocilizumab, attention must be paid to the well-known risk factors for MRONJ. Rheumatologists should also be involved in treatment decisions since autoimmune diseases are common comorbidities. 

## Notes

### Ethical statement

This research was conducted in full accordance with ethical principles, including the World Medical Association Declaration of Helsinki. The patients’ data were referenced to with the understanding and written consent of the patient, and all data were also anonymized and de-identified prior to analysis. Reporting was based on the recommendations of the Strengthening the Reporting of Observational Studies in Epidemiology (STROBE) initiative [32].

### Competing interests

The authors declare that they have no competing or financial interests, either directly or indirectly, in the products listed in the study.

### Acknowledgments:

We would like to thank Bacon Editing^®^ for language editing of this manuscript.

## Figures and Tables

**Table 1:**
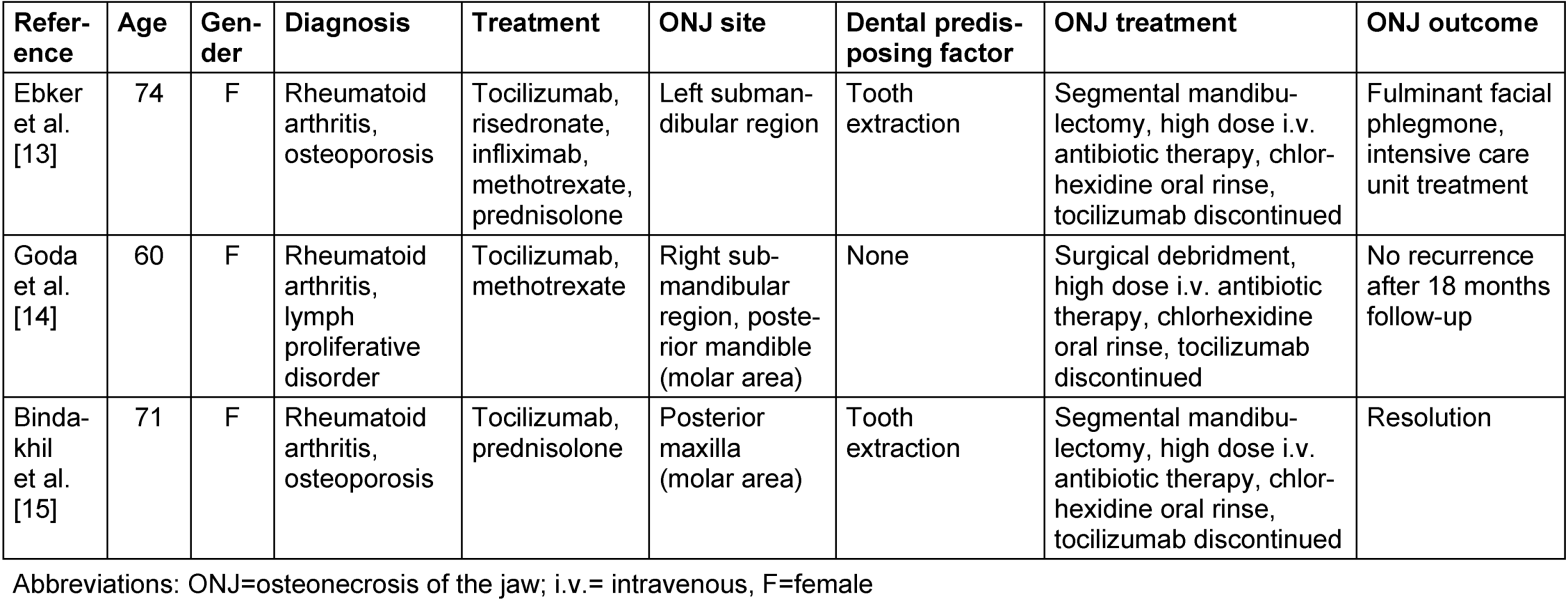
Previously reported cases of tocilizumab-related osteonecrosis of the jaws (with or without co-treatment with bisphosphonates or other drugs)
